# PredMHC: An Effective Predictor of Major Histocompatibility Complex Using Mixed Features

**DOI:** 10.3389/fgene.2022.875112

**Published:** 2022-04-25

**Authors:** Dong Chen, Yanjuan Li

**Affiliations:** College of Electrical and Information Engineering, Quzhou University, Quzhou, China

**Keywords:** protein classification, major histocompatibility complex, machine learning, feature extraction, identification

## Abstract

The major histocompatibility complex (MHC) is a large locus on vertebrate DNA that contains a tightly linked set of polymorphic genes encoding cell surface proteins essential for the adaptive immune system. The groups of proteins encoded in the MHC play an important role in the adaptive immune system. Therefore, the accurate identification of the MHC is necessary to understand its role in the adaptive immune system. An effective predictor called PredMHC is established in this study to identify the MHC from protein sequences. Firstly, PredMHC encoded a protein sequence with mixed features including 188D, APAAC, KSCTriad, CKSAAGP, and PAAC. Secondly, three classifiers including SGD, SMO, and random forest were trained on the mixed features of the protein sequence. Finally, the prediction result was obtained by the voting of the three classifiers. The experimental results of the 10-fold cross-validation test in the training dataset showed that PredMHC can obtain 91.69% accuracy. Experimental results on comparison with other features, classifiers, and existing methods showed the effectiveness of PredMHC in predicting the MHC.

## Introduction

As a large locus on vertebrate DNA, the major histocompatibility complex (MHC) contains a tightly linked set of polymorphic genes encoding cell surface proteins that are essential for immune surveillance. These cell surface proteins are called MHC molecules (Kubiniok et al., 2022). MHC molecules are classified into MHC class I, MHC class II, and MHC class III according to variation in molecular structure, function, and distribution (Marcoux et al., 2021). MHC class I molecules are expressed in all nucleated cells and platelets—essentially all cells except red blood cells, which display antigens to signal cytotoxic T lymphocytes, including clusters of differentiation (CD8^+^) (McShan et al., 2021). MHC class II molecules are expressed in antigen-presenting cells, such as B cells, dendritic cells, and macrophages, where they normally bind to CD4^+^ receptors on helper T cells to clear foreign antigens. MHC class III genes are interleaved with class I and class II genes on the short arm of chromosome 6, but their proteins play different physiological roles.

MHC molecules are cell surface glycoproteins with a three-dimensional structure and are of vital importance to infection, autoimmunity, transplantation, and tumor immunotherapy. MHC-binding prediction plays an important role in identifying potential novel therapeutic strategies. Mahoney et al. (2021) pointed out that MHC phosphopeptides can be considered potential immunotherapeutic targets for cancer and other chronic diseases. Therefore, many scholars carried out a lot of research work on MHC-binding prediction. The first computational method (Altuvia et al., 1995) to uncover the MHC-binding peptide was developed by Altuvia et al., which is based on protein structure and is further improved to distinguish candidate peptides that bind to hydrophobic binding pockets of the MHC molecules (Altuvia et al., 1997). The SVRMHC (Liu et al., 2006) is an MHC-binding peptide model which encoded peptides with physicochemical properties and trained support vector machines to construct a prediction model on mice. NetMHC-3.0 (Lundegaard et al., 2008) is a web server with high performance for predicting peptide binders based on artificial neural networks. Boehm et al. proposed a method named ForestMHC (Boehm et al., 2019) to identify immunogenic peptides. ForestMHC encoded a peptide sequence with physicochemical properties and trained a random forest classifier to construct an identification model. Saxena et al. (2020) predicted the binding potential of peptides to the MHC, which is critical for designing peptide-based therapeutics, using a deep learning model named OnionMHC. In consideration of the importance of structural information, the OnionMHC represents peptides with its sequence and structure-based features for peptide-HLA-A*02:01 binding predictions. (Lv et al., 2020) Jiang et al. (2021) gave a comprehensive review of the state-of-the-art literature on MHC-binding peptide prediction and an in-depth evaluation of feature representation methods, prediction models, and model training strategies on benchmark datasets. Based on the limitation of only handling peptide sequences with fixed length, Jiang et al. proposed a novel variable-length MHC-binding prediction model named BVLSTM-MHC. Experimental results on an independent validation dataset showed that BVLSTM-MHC has better performance than the ten mainstream prediction tools.

Scientists are devoted to discover MHC molecules in various vertebrate genomes. Hopkins et al. (1986) described a rat monoclonal antibody which can recognize MHC class II antigens in sheep and seems to recognize determinants which are nonpolymorphic. Moreover, based on the antibody, the distribution of sheep class II molecules is investigated, and the class II- expression variations by cells in efferent lymph and peripheral is also investigated. Westbrook et al. (2015) combined the SMRT sequencing technology and CCS and introduced and validated the technology of SMRT-CCS on identifying class I transcripts in Mauritian-origin cynomolgus macaques. Furthermore, SMRT-CCS was applied to characterize 60 new full-length class I transcriptional sequences expressed in the Chinese cynomolgus monkey population. By using pyrosequencing with high-resolution and Sanger sequencing technology, Shiina et al. (2015) genotyped 127 unrelated animals and identified 112 different alleles. Moreover, the International Society for Animal Genetics (ISAG) standardized the nomenclature and established the IPD-MHC database which is used to scientifically manage the MHC allele sequences and genes from nonhuman organisms (Giuseppe et al., 2017; Maccari et al., 2018; Ali et al., 2021; Burton et al., 2021; Karcioglu and Bulut, 2021; Roy et al., 2021; Safaei et al., 2021; Wang et al., 2021).

At early stages, the research studies related to the MHC are developed based on mice experiments. With the availability of a large amount of data and development of machine learning, developing a machine learning–based model to research the MHC was feasible. Li et al. (2019) proposed an identification method of the MHC based on an extreme learning machine algorithm. Although high accuracy has been achieved, there are still many aspects worthy of further investigation (Lv et al., 2019; Lv et al., 2021a; Lv et al., 2021b). In this study, we aim to propose a new MHC predictor, PredMHC, to further improve prediction performance.

## Materials and Methods

### Framework of PredMHC

**FIGURE 1:**
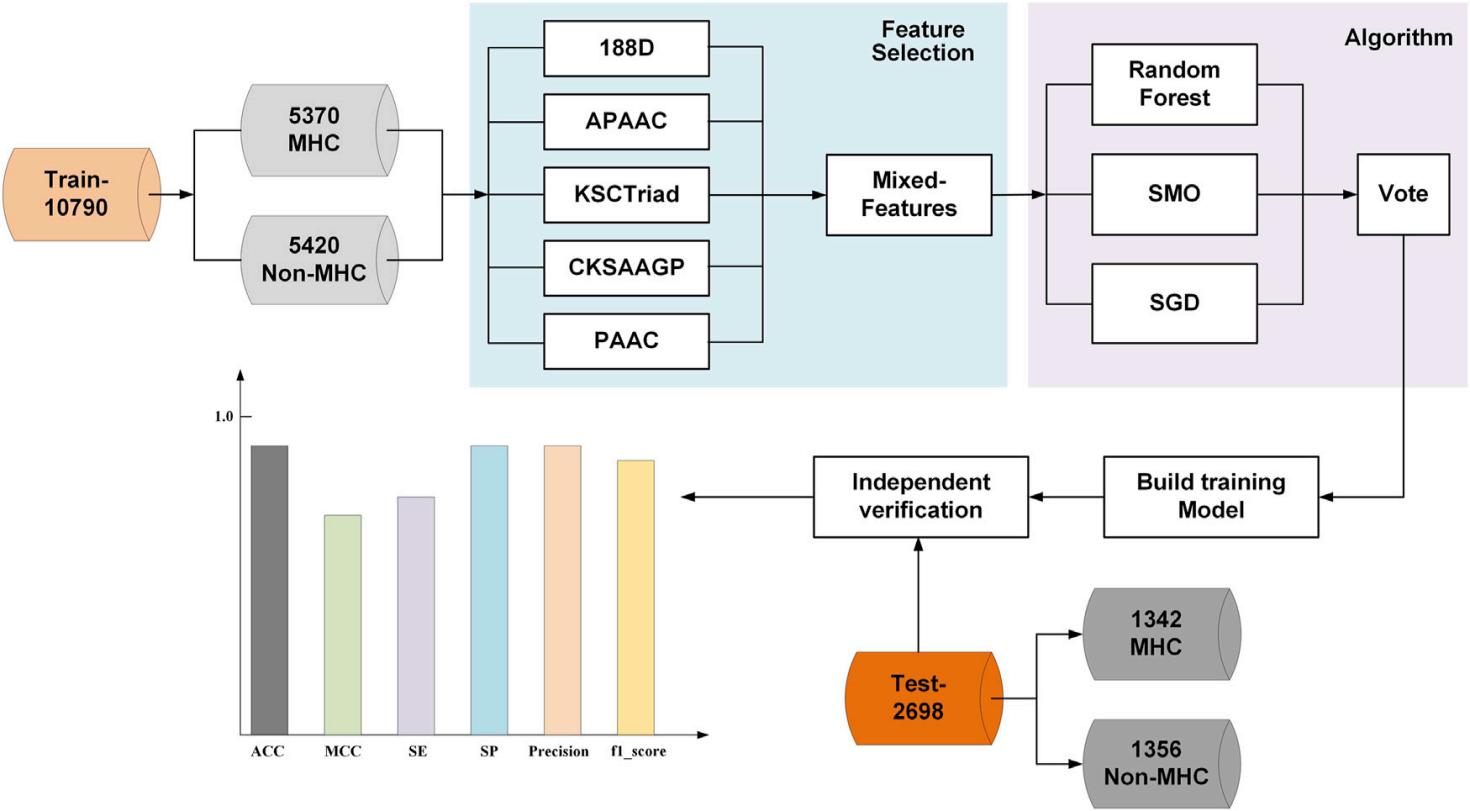
Framework of PredMHC.

In this study, we introduced a novel MHC predictor named PredMHC, the framework of which is shown in Figure 1. First, PredMHC encoded a protein sequence with mixed features including 188D, APAAC, KSCTriad, CKSAAGP, and PAAC. Second, three classifiers including SGD, SMO, and random forest were trained on the mixed features of protein sequence. Finally, the prediction result was obtained by the voting of the three classifiers. We will introduce the datasets, feature extraction, and classifiers in detail in the following section.

### Dataset

The dataset constructed by Li et al. (2019) is used in this study. A web server called ELM-MHC was developed by Li et al., from which the dataset can be downloaded. The reason that we used the same dataset as ELM-MHC is as follows. First, the dataset is constructed by searching for MHC sequences on the Uniprot database, and it is reliable. Second, the dataset is used cd-hit to de-duplication processing. The protein sequences are clustered based on the parameter setting, and the sequence with the maximum length in every cluster is used as a representative sequence. The redundant and homology-biased sequences are removed in this dataset. Finally, the most important inference was that we can fairly compare with the existing method by using the same dataset. The final dataset contained 13,488 protein sequences, which consists of 6,712 MHC protein sequences (positive examples) and 6,776 nonMHC protein sequences (negative examples). All protein sequences were divided into two groups: 10,790 sequences as a set of 10-fold cross-validation and 2,698 sequences as a set of independent validation. The training dataset (Train-10790) comprised 5,370 MHC protein sequences and 5,420 nonMHC protein sequences, all randomly selected from the set of positive and negative examples, respectively. They were then further randomly divided into five sets for the input of 10-fold cross-validation. The independent testing dataset (Test-2698) contained 1,342 positive and 1,356 negative examples.

### Feature Extraction

To classify a protein sequence into different categories using the machine learning method, the first step is to encode the protein sequence with features. A feature that can effectively discriminate positive examples from negative examples can greatly improve the prediction performance of the model. In this study, we try to encode protein sequences with mixed features including 188D, APAAC, KSCTriad, CKSAAGP, and PAAC. The mixed features can represent a protein sequence from different prospectives; thus, it can better distinguish different protein sequences.

#### SVMProt-188D

SVMProt-188D is a feature extraction method based on the amino acid composition and physicochemical properties (Dubchak et al., 1995; Saxena et al., 2021). It encodes each protein sequence as a 188-dimensional feature vector. The first 20 features are the frequencies of the 20 amino acids (A, C, D, E, F, G, H, I, K, L, M, N, P, Q, R, S, T, V, W, and Y in alphabetical order) occurring in the sequence. The formula is defined as

$$\left(V_{1},\;\;V_{2},\;\;\;...,\;V_{20}\right)=\frac{N_{i}}{L},$$

where *N*_i_ denotes the number of the *i*th amino acid in the protein sequence and L denotes the length of a sequence. Obviously, $\sum V_{i}=1$ .

The latter dimensions are correlated with eight physicochemical properties, namely, hydrophobicity, normalized Van der Waals volume, polarity, polarizability, charge, surface tension, secondary structure, and solvent accessibility. Each physicochemical property consists of 21 numbers. In detail, each property consists of three descriptors, composition (C), transition (T), and distribution (D). C indicates the proportion of amino acids with specific physicochemical properties to all amino acids, and the dimension of C is 3; T represents the percentage frequency of amino acids with a specific property behind amino acids with another property, and its dimension is 3; and D represents the proportions of the chain length of 0, 25, 50, 75, and 100% amino acids with a specific property, and its dimension is 8. Therefore, after analyzing the composition and eight physicochemical properties of amino acids, we can obtain a total of 20+(3 + 5+8)×8 = 188 features.

#### Amphiphilic Pseudo Amino Acid Composition

The concept of amphiphilic pseudo amino acid composition (APAAC), originally proposed by Chou (Chou, 2005; Lv et al., 2021a; Awais et al., 2021; Naseer et al., 2021; Yan et al., 2021), is an effective protein descriptor and has been applied for diverse protein sequence analysis. APAAC is different from traditional AAC. It can incorporate a partial sequence-order effect by using the hydrophobicity and hydrophilicity of the constituent amino acids in a protein. For the convenience of the readers, we will briefly introduce the concept of APAAC. Let R_1_R_2_R_3_...R_L_ be a protein sequence with length L, where R_1_ denotes the residue at position 1, R_2_ denotes the residue at positon 2, and so forth. According to the definition of APAAC, a protein can be denoted as a vector P with dimension (20+2λ). Vector P is defined as follows.

$$P=\left[P_{1},\ldots ,P_{20},P_{20+1},\ldots ,P_{20+\lambda },\ldots ,P_{20+2\lambda }\right],$$ (1)

where P_1,_ P_2_, … , P_20_ in Eq. 1 represent the classic AAC and the next 2λ discrete numbers describe the sequence correlation factor.

#### K-Spaced Conjoint Triad

The k-spaced conjoint triad (KSCTriad) (Chao et al., 2018; Zhen et al., 2020) is an effective protein descriptor and has been comprehensively applied for diverse biological sequence analyses. Different from the conjoint triad descriptor, KSCTriad not only calculates the number of three continuous amino acid units but also incorporates the continuous amino acid units that are separated by any k-residues.

#### Composition of K-Spaced Amino Acid Group Pairs

The composition of k-spaced amino acid pairs (CKSAAP) (Chen et al., 2010; Ahmad et al., 2021; Akbar et al., 2021; Al-Qazzaz et al., 2021; Alar and Fernandez, 2021; Alim et al., 2021; Buriro et al., 2021) method describes the order-related information of the protein sequence, which takes the occurrence frequency of two amino acids separated by k-residues in the sequence as a feature element. The protein contains 20 amino acids; thus, a 400-dimensional feature vector can be obtained for each interval. The composition of k-spaced amino acid group pairs (CKSAAGP) is a variation of the CKSAAP method. The 20 amino acids can be classified into five groups based on the chemical properties of their side chains: the aliphatic group, aromatic group, positive charged group, negative charged group, and uncharged group. The CKSAAGP method is based on the frequency of the two groups separated by a k-spaced amino acid.

#### Pseudo-Amino Acid Composition

The conventional amino acid composition is defined in a 20-D space, and each dimension represents the frequency of the occurrence of one of the 20 native amino acids. Different from the conventional amino acid protein composition, the pseudo-amino acid composition (Chou, 2001; Awais et al., 2021), which is a vector with 20+λ discrete components, will contain much more sequence-order and sequence-length information. According to the concept of pseudo-amino acid composition, the feature is given by

$$P=\left[\begin{array}{c} p_{1}\\ \vdots \\ p_{20}\\ p_{20+1}\\ \vdots \\ p_{20+\lambda } \end{array}\right],$$

where the first 20 components are the occurrence frequencies of the 20 amino acids in the protein which is the same as in the conventional amino acid composition, while the additional components p_20+1_ … p_20+λ_ are the sequence-order correlation factors of the different ranks.

### Classifier

To obtain better classification results, we adopted the voting of three base classifiers as the final classification result. The three classifiers were, respectively, random forest, SMO, and SGD. The three classifiers are popular and have been successfully used in bioinformatics many times.

Random forest is an ensemble classifier based on the decision tree algorithm proposed by Breiman in 2001 (Breiman, 2001). To solve regression or classification tasks, random forests construct many decision trees by extracting subsets from all the samples through the bootstrap technique and obtain the prediction result by voting on these decision trees. Random forests are widely used in bioinformatics because of their low computational overhead and ability of handling unbalanced data.

The support vector machine (SVM) (Hearst et al., 1998) is a well-known machine learning algorithm that completes various classification tasks by constructing a separating hyperplane in the high-dimensional space. However, the training speed of support vector machines is heavily influenced by data size. To solve this problem, the sequential minimum optimization (SMO) (Platt, 1999) algorithm was proposed, which decomposes large quadratic programming problems (OPs) of an original SVM into a series of the smallest possible QP problems. Moreover, the solution process of SMO needs no additional matrix storage, thus saving both time and space costs.

The goal of the stochastic gradient descent (SGD) algorithm is to find a path that leads to optimal result. When using this algorithm, the parameter values are first initialized, and then these values are continuously changed until the target function converges. The SGD algorithm is widely used to process large-scale sparse data, such as text classification tasks.

### Measurement

To evaluate the performance of the proposed method, we introduced four indicators commonly used in bioinformatics: sensitivity (SE), specificity (SP), accuracy (ACC), and Matthew’s correlation coefficient (MCC). The formulae of these indicators are as follows (Zhang et al., 2021a; Lv et al., 2021b; Zhang et al., 2021b; Zhang et al., 2021c; Zhang et al., 2021d; Zhang et al., 2021e; Zhao et al., 2021; Zhu et al., 2021; Zou et al., 2021; Zhao et al., 2022).

$$SE=\frac{TP}{TP+FN},$$

$$SP=\frac{TN}{TN+FP},$$

$$ACC=\frac{TN+TP}{TN+FP+TP+FN},$$

$$MCC=\frac{\left(TP\times TN\right)-\left(FP\times FN\right)}{\sqrt{\left(TP+FP\right)\times \left(TP+FN\right)\times \left(TN+FP\right)\times \left(TN+FN\right)}},$$

where TP is an abbreviation for true positives, representing the number of MHC proteins predicted in positive examples; FP is an abbreviation for false positives, representing the number of MHC proteins predicted in negative examples; TN is an abbreviation for true negatives, representing nonMHC proteins predicted in negative examples; and FN is an abbreviation for false negatives and indicates the number of predicted nonMHC proteins in positive examples. SE and SP represent the predictive accuracy of the model in positive and negative samples, respectively. Both ACC and MCC represent the overall performance of the model. For all the aforementioned metrics , the higher the score they get the better the performance of the model.

## Result and Discussion

### Cross-Validation Results of Train-10790

In many experiments, we tried a variety of methods to extract highly recognizable features from protein sequences in the training set and used several algorithms to train the model to achieve optimal accuracy. The experimental comparison results of different features are explained in *Performance of Different Features on Cross-Validation*, and the experimental comparison results of different classifiers are explained in *Performance of Different Classifiers on Cross-Validation*.

#### Performance of Different Features on Cross-Validation

Using the voting of random forest, SMO, and SGD as the classification model, we first tried 188D, APAAC, KSCTriad, CKSAAGP, PAAC, and their combinations. Table 1 shows the performance of the five single features and several combinations of features with good performance in the 10-fold cross-validation. As shown in Table 1, according to the indexes MCC and ACC, the mixed features proposed in this study have the highest score; thus, our method has better overall performance. According to the indicator of SE, the feature of APAAC has the highest score, whereas its value of ACC, MCC, and SP is lower; it verifies that the feature of APAAC was bias to classify a protein into the MHC protein. Similar to APAAC, PAAC also has higher value on the indicator SE and lower value on other indicators. Therefore, from the overall perspective, our method obviously performs better than all other methods.

**TABLE1:** Result of different features on Train-10790.

Feaures	ACC	MCC	SE	SP
(1)-188D	0.8953	0.7927	0.8596	0.9310
(2)-APAAC	0.8329	0.6824	0.9494	0.7108
(3)-KSCTriad	0.8764	0.7580	0.8177	0.9350
(4)-CKSAAGP	0.8682	0.7469	0.7826	0.9529
(5)-PAAC	0.8283	0.6739	0.9485	0.7018
188D + APAAC	0.9003	0.8019	0.8735	0.9276
APAAC + KSCTriad	0.8872	0.7782	0.8386	0.9360
KSCTriad + CKSAAGP	0.8993	0.8039	0.8404	0.9576
CKSAAGP + PAAC	0.8848	0.7728	0.8376	0.9316
188D + APAAC + KSCTriad	0.9121	0.8268	0.8734	0.9511
APAAC + KSCTriad + CKSAAGP	0.9054	0.8155	0.8518	0.9589
KSCTriad + CKSAAGP + PAAC	0.9041	0.8127	0.8516	0.9565
188D + APAAC + KSCTriad + CKSAAGP	0.9157	0.8351	0.8701	0.9618
APAAC + KSCTriad + CKSAAGP + PAAC	0.9065	0.8178	0.8522	0.9608
Our mixed feature	0.9169	0.8370	0.8761	0.9587

#### Performance of Different Classifiers on Cross-Validation

To verify the performance of our used classifier, we compared the classifier used in this study with other classifiers. Table 2 shows the experimental results. As shown in Table 2, the voting of SGD, SMO, and random forest used in our identification system has better performance than other single classifiers. As shown in Table 2, our classification model has 0.9169% accuracy and 0.8370 MCC, which are higher than those of other classifiers. It verified that our classification model has better overall performance. According to the number of winning incidences, our classification wins on three indicators and has the highest number of wins. It is shown in Table 2 that the SE of our classification model was slightly lower than that of random forest. However, the values of ACC, MCC, and SP of our classification model are obviously higher than those of random forest. Therefore, from the overall perspective, our classification model obviously performs better than all other classifiers.

**TABLE 2:** Result of different classifiers on Train-10790.

Classifiers	ACC	MCC	SE	SP
SGD	0.8794	0.7600	0.8504	0.9081
SMO	0.9038	0.8106	0.8594	0.9478
Random forest	0.8850	0.7699	0.8830	0.8869
Our classification model	0.9169	0.8370	0.8761	0.9587

### Independent-Validation Results of Test-2698

To evaluate the generalization performance of the proposed model, we tested its performance on the Test-2698 dataset. In detail, we trained the model proposed in this study on the Train-10790 dataset and then computed its performance on the test-2698 dataset. The experimental results are shown in Tables 3, 4. As shown in Tables 3, 4, the feature extraction method and classifier used in this study have better performance than the other feature extraction methods and classifiers, respectively.

**TABLE 3:** Result of different features on Test-2698.

Features	ACC	MCC	SE	SP
188D	0.8926	0.7869	0.8593	0.9259
APAAC	0.8357	0.6892	0.9533	0.7139
KSCTriad	0.8741	0.7504	0.8355	0.9127
CKSAAGP	0.8774	0.7614	0.8098	0.9442
PAAC	0.8326	0.6826	0.9527	0.7056
188D + APAAC	0.9010	0.8061	0.8482	0.9530
APAAC + KSCTriad	0.8940	0.7888	0.8697	0.9182
KSCTriad + CKSAAGP	0.9055	0.8155	0.8540	0.9573
CKSAAGP + PAAC	0.8901	0.7818	0.8571	0.9230
188D + APAAC + KSCTriad	0.9172	0.8355	0.8938	0.9412
APAAC + KSCTriad + CKSAAGP	0.9130	0.8287	0.8729	0.9532
KSCTriad + CKSAAGP + PAAC	0.9155	0.8337	0.8769	0.9544
188D + APAAC + KSCTriad + CKSAAGP	0.9198	0.8416	0.8841	0.9550
APAAC + KSCTriad + CKSAAGP + PAAC	0.9134	0.8300	0.8693	0.9574
Our mixed feature	0.9246	0.8502	0.9034	0.9466

**TABLE 4:** Result of different classifiers on Test-2698.

Classifier	ACC	MCC	SE	SP
SGD	0.8959	0.7918	0.8935	0.8982
SMO	0.9063	0.8147	0.8682	0.9440
Random forest	0.8948	0.7896	0.8913	0.8982
Our classification model	0.9246	0.8502	0.9034	0.9466

### Comparison With Other Predictors

To evaluate the performance of the classifier PredMHC, we compared it with ELM-MHC on the same dataset including Train-10790 and Test-2698. The comparison results on the 10-fold cross-validation are shown in Table 5. As we can see from Table 5, PredMHC has higher score than ELM-MHC on the indicators ACC, MCC, and SP. According to the number of winning incidence, PredMHC has better performance than ELM-MHC. According to ACC and MCC, PredMHC has better overall performance than ELM-MHC. Therefore, PredMHC is superior to the existing methods in the prediction of MHC protein.

**TABLE 5:** Comparison of 10-fold cross-validation with the existing method on all data.

Method	ACC	MCC	SE	SP
ELM-MHC	0.9166	0.822	0.893	0.908
Our method	0.9185	0.8403	0.8741	0.9627

## Conclusion

In this study, we proposed an efficient, reliable, and simple experimental model for predicting the MHC protein based on mixed features. After a large number of comparative experiments, we selected the mixed features of 188D, APAAC, KSCTriad, CKSAAGP, and PAAC, which showed global performance on the 10-fold cross-validation training dataset and independent test dataset. We then used the voting of SGD, SMO, and random forest to build a prediction model which also achieved the best performance on both training and test datasets. In terms of important indicators, our model obtained an MCC of 0.8370 and ACC of 0.9169 in the 10-fold cross-validation based on the Train-10790 dataset and MCC of 0.8502 and ACC of 0.9246 in the independent validation based on the Test-2698 dataset. In conclusion, we believe that our novel model provides an efficient and reliable method to screen MHCs from a large number of protein sequences. In the future, we will pay more attention to deep learning classifiers and evolution strategies (Tahoces et al., 2021; Tandel et al., 2021; Tavolara et al., 2021; Togacar, 2021; Tsiknakis et al., 2021; Turki and Taguchi, 2021; Usman et al., 2021; Vafaeezadeh et al., 2021; Wang et al., 2021; Watanabe et al., 2021; Yap et al., 2021; Yildirim et al., 2021).

## Data Availability

The original contributions presented in the study are included in the article/Supplementary Material, further inquiries can be directed to the corresponding author.
